# Diagnosis and management of paediatric *Nocardia* keratitis: a case report

**DOI:** 10.1093/omcr/omag092

**Published:** 2026-06-08

**Authors:** Constantinos Efthyvoulou, Kiki Savvidou, Naso Theodorou, Esther Papamichael

**Affiliations:** Imperial College School of Medicine, Faculty of Medicine, Imperial College London, Exhibition Road, London SW7 2AZ, United Kingdom; Bioanalysis Medical Laboratory Centre, Kyriakou Matsi and Glafkou 1, Office 101, 1085, Nicosia, Cyprus; Bioanalysis Medical Laboratory Centre, Kyriakou Matsi and Glafkou 1, Office 101, 1085, Nicosia, Cyprus; IRIS Ophthalmology Centre, Esperidon 15-3rd floor, Strovolos 2001, Nicosia, Cyprus

**Keywords:** *Nocardia* species, *Nocardia* keratitis, paediatric keratitis, infectious keratitis

## Abstract

*Nocardia* species are Gram-positive, aerobic actinomycetes. *Nocardia* keratitis is a rare ocular infection that is characterised by an atypical presentation and may be unresponsive to empirical antibiotic therapies. We report a case of *Nocardia* keratitis in a 10-year-old female patient caused by a corneal injury from a palm tree thorn marked by a delayed diagnosis and challenging management course. Initially, her visual acuity was counting fingers and treatment with moxifloxacin was ineffective. Multiple cultures returned negative for growth of pathogens, delaying the diagnosis of *Nocardia* species until day 32 from presentation. Once diagnosed, an aggressive antibiotic regimen was initiated. To address suboptimal control of infection and minimise the burden of frequent drops, an intrastromal ceftazidime injection was administered. Eventually, complete resolution and restoration of visual acuity were achieved. *Nocardia* keratitis is challenging to diagnose and requires prolonged treatment; however, early suspicion and appropriate management are associated with good prognosis and favourable outcomes.

## Introduction

**Figure 1:**
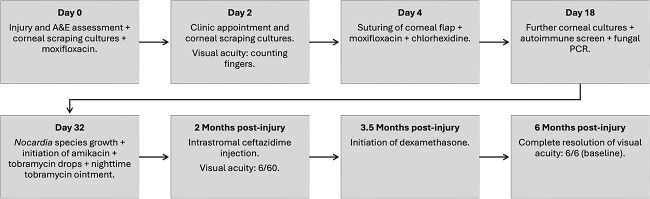
Timeline of diagnosis and management from the day of injury.

*Nocardia* keratitis is a rare ocular condition reported globally, with a higher prevalence in Asia [1, 2]. In Asian countries, most cases are due to corneal injuries, particularly in rural areas, whereas in the United States, contact lens misuse is the predominant risk factor [3]. The prevalence of *Nocardia* among all cases of bacterial keratitis in Asia varies from 0.3% to 4.2% [4]. Elsewhere, the true prevalence is largely unknown, with sporadic reports of the condition [1, 3]. This report aims to explore the diverse presentation of *Nocardia* keratitis, enhancing clinicians' ability to recognise signs that may raise its suspicion, potentially preventing misdiagnosis. Additionally, it outlines the management employed in a paediatric case that led to an optimal clinical outcome (Fig. 1).

## Case report

A previously healthy 10-year-old female patient sustained a left ocular trauma from a palm tree thorn. She was assessed in the emergency department the same day, corneal scraping cultures were obtained, and she was discharged home with prophylactic moxifloxacin 0.5% drops.

**Figure 2:**
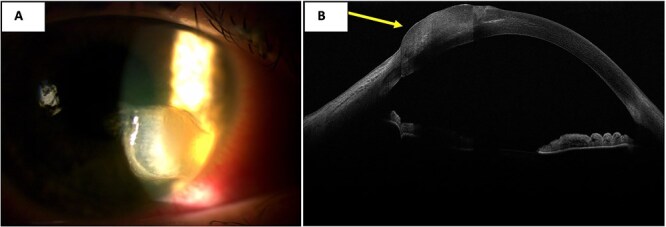
(A) Corneal flap caused by a partial thickness, non-penetrating laceration. (B) Anterior chamber optical coherence tomography (OCT) from day 2. Arrow indicates partial thickness non-penetrating corneal flap.

She presented to our clinic with worsening pain, photophobia, blurred vision and discharge from her left eye. On examination, her left eye appeared injected and inflamed with an oedematous corneal flap caused by a partial thickness, non-penetrating laceration (Fig. 2). Visual acuity was 6/6 in the right eye and counting fingers in the left. Further cultures were obtained and moxifloxacin was continued.

**Figure 3:**
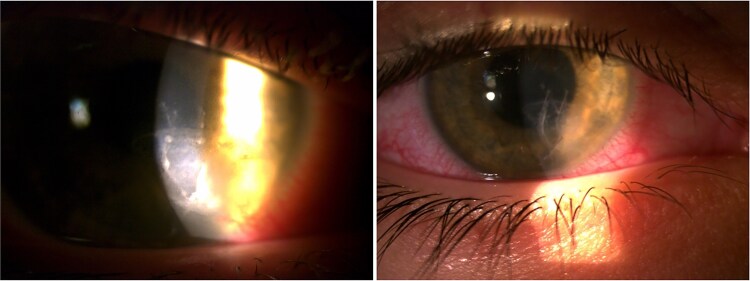
Sutured corneal flap visualised under slit-lamp examination.

Once the cultures yielded no growth of any pathogen, the injury was debrided and sutured under general anaesthesia with new cultures obtained from deep within the flap (Fig. 3). Postoperative treatment included moxifloxacin 0.5% and chlorhexidine 0.2%. However, visual acuity remained suboptimal at 6/60.

Two weeks post-injury, cultures yielded no valuable information. A loose suture was removed and sent for further cultures. Central corneal melting prompted an autoimmune and immunodeficiency screen, which were negative. Due to feathery-like infiltrates, a fungal PCR test was also performed.

**Figure 4:**
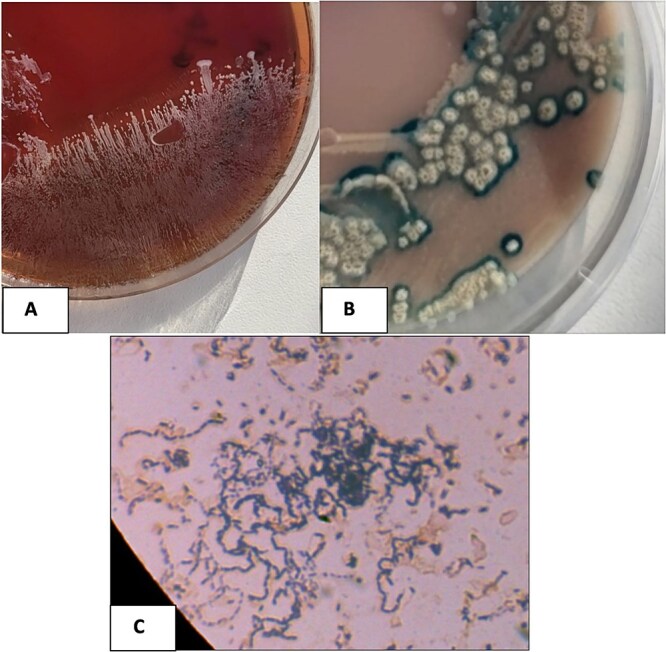
(A) Growth of *Nocardia* species colonies on Columbia blood agar. (B) Growth of *Nocardia* species colonies on Sabouraud dextrose agar. (C) Microscopic examination of *Nocardia*, isolated from our specimen, showing gram-positive bacteria with pronounced polymorphism. They exhibit characteristic branching filaments that stain irregularly, as well as coccoid or coccobacillary forms. The diameter of their cells is approximately 1 μm.

After cessation of all treatment, further cultures were obtained, and on day 32 they revealed growth of *Nocardia,* leading to the diagnosis of *Nocardia* keratitis (Fig. 4). Aggressive treatment was initiated with hourly drops of amikacin 2.5%, tobramycin 0.3%, and a night-time ointment of tobramycin. After one month, visual acuity improved to 6/24, though still suboptimal. To address compliance issues from the burden of hourly drops, a 0.4 mL intrastromal ceftazidime 22.2 mg/ml injection was administered. Additionally, topical treatment with polymyxin B sulfate-trimethoprim 10 000 units/ml, 1 mg/ml drops (previously unavailable), and cyclosporine 0.1% were prescribed.

**Figure 5:**
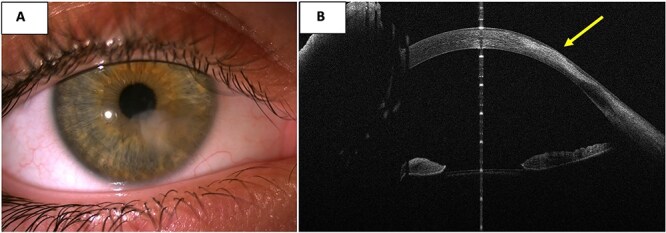
(A) Corneal scar 6 months after complete resolution and restoration of visual acuity. (B) Arrow shows corneal scar on anterior optical coherence tomography (OCT) after 6 months, demonstrating epithelial restoration.

Approximately 3.5 months post-injury, with the infection controlled, dexamethasone 0.1% drops were initiated. At 6 months, the patient had no permanent vision loss with 6/6 visual acuity in both eyes (Fig. 5).

## Discussion

*Nocardia* species are aerobic actinomycetes found in soil, standing water and decaying plants [5]. They are rod-shaped, gram-positive, catalase-positive opportunistic bacteria. *Nocardia* can resemble fungi or nontuberculous mycobacteria, forming acid-fast staining filaments; nonetheless, despite their morphology, they are classified as true bacteria [5]. They are slow-growing, typically forming dry, chalky colonies taking up to 14 days [5]. In this case, colonies appeared after 4 days of aerobic incubation at 35–37°C on Columbia blood and Sabouraud dextrose agar (Fig. 4). Additionally, the characteristic Gram stain was observed during microscopic examination (Fig. 4). While the typical slit lamp presentation of *Nocardia* keratitis features patchy corneal infiltrates in a wreath-like pattern, this was not observed in our case [6].

Several factors contributed to the delayed diagnosis of *Nocardia*. Its atypical presentation, lacking characteristic features, hindered clinical suspicion. Furthermore, initial antibiotic therapy may have inhibited microbial growth in subsequent cultures, while failing to eradicate the infection in vivo. The slow-growing nature of *Nocardia* further complicated its detection, potentially leading to early false-negative reports. To address this, clinicians should notify the laboratory of suspected *Nocardia* to extend the incubation period [6]. In response to the persistent, non-resolving infection, microbiological investigations were escalated, including additional cultures and fungal PCR. In vivo confocal microscopy may aid in early detection, revealing thin filaments; however, it was not available locally [6].

*Nocardia* infections often fail to respond to first-line empirical antibiotics. Topical amikacin 2.5% monotherapy is considered the gold-standard treatment against *Nocardia* species [4]. Although our isolate was fully susceptible, amikacin resistance has been documented in other reports. A study by Adre *et al*. from the United States revealed amikacin resistance in 64% of cases [3]. Notably, all isolates demonstrated in-vitro susceptibility to Trimethoprim-sulfamethoxazole.

A study by Lalitha *et al.* showed the highest recovery rates in patients presenting within 15 days of infection [7]. Given the delayed identification of the pathogen (day 32), combination antibiotic therapy was employed to leverage a synergistic effect, avoiding further delays in effective treatment [4]. An intrastromal ceftazidime injection was administered to address compliance issues due to the burden of hourly drops. Evidence suggests intrastromal injections enhance resolution of non-resolving infectious keratitis by increasing local antibiotic concentration [8]. However, standardised guidelines for antibiotic selection and dosage are limited. The procedure carries risks, such as infection spread, hyphema and intrastromal bleeding; although, these are rare [8]. Additionally, general anaesthesia is required for paediatric patients.

Studies demonstrated that corticosteroids are contraindicated in active *Nocardia* keratitis due to risk of reinfection and worsened clinical outcomes [9, 10]. Given the significant inflammation, cyclosporine was used to control the immune response, aiming to limit scarring. Corticosteroids were initiated only after pathogen eradication, with antibiotic coverage and close monitoring, as the benefits of symptom control and scar prevention were deemed to outweigh the risks. Associated risks with their use include delayed healing and corneal perforation [10].

Although rare outside Asia, *Nocardia* keratitis is documented globally. This case highlights the importance of considering *Nocardia* as a differential diagnosis in non-resolving keratitis mimicking a fungal infection. In paediatric cases, compliance awareness may necessitate alternative antibiotic delivery, such as intrastromal injections. Early recognition of both typical and atypical presentations is key to avoiding misdiagnosis, ensuring effective treatment and visual outcomes. Although a single case report has limited generalisability, it reinforces the need for a high index of suspicion for *Nocardia* and may guide management in similar scenarios.
